# NRP1 silencing induces ROS-mediated cell cycle arrest and mitochondrial apoptosis in non-small cell lung cancer via upregulation of AGER

**DOI:** 10.1016/j.clinsp.2026.101080

**Published:** 2026-08-13

**Authors:** Ya Yang, Ying Li, Yongjuan Zhao, Wenwen Feng, Pengcheng Yu, Shan Li, Zongli Ding

**Affiliations:** a Department of Geriatrics, The Affiliated Huai’an Hospital of Xuzhou Medical University, Huai’an, Jiangsu Province, China; b Xuzhou Medical University, Jiangsu Province, China

**Keywords:** NRP1, AGER, Lung cancer, Oxidative stress, Apoptosis

## Abstract

**Objective:**

The specific role of Neuropilin-1 (NRP1) and its link to oxidative stress in Non-Small Cell Lung Cancer (NSCLC) progression remains unclear.

**Methods:**

Integrated bioinformatics, including weighted gene co-expression network analysis and machine learning, were applied to the TCGA cohort and validated in an age-stratified clinical cohort. In vitro assays evaluated proliferation, apoptosis, ATP, and Reactive Oxygen Species (ROS) in NRP1-silenced H1299 cells. Mechanisms were investigated using endogenous Co-Immunoprecipitation (Co-IP) and rescue experiments with AGER siRNA and the ROS scavenger N-Acetylcysteine (NAC).

**Results:**

Bioinformatics identified AGER as a key NRP1 downstream target. Clinically, NRP1 was significantly upregulated exclusively in elderly (≥65-years) NSCLC patients, whereas AGER was consistently downregulated. Functionally, NRP1 silencing inhibited proliferation, induced G1-phase arrest, and triggered mitochondria-dependent apoptosis characterized by severe ATP depletion and ROS accumulation. Endogenous Co-IP confirmed a direct physical interaction between NRP1 and AGER. Importantly, the oxidative stress and apoptotic phenotypes induced by NRP1 knockdown were effectively reversed by AGER silencing or NAC treatment.

**Conclusion:**

The NRP1-AGER-ROS axis critically regulates tumor progression and oxidative stress in NSCLC, particularly in elderly patients, representing a promising age-associated therapeutic target.

## Highlights

• NRP1 is significantly upregulated, whereas AGER is downregulated in NSCLC tissues.
• Silencing NRP1 inhibits NSCLC cell proliferation and induces G1 phase arrest.
• NRP1 knockdown triggers mitochondria-dependentk apoptosis and ROS accumulation.
• AGER overexpression suppresses cell growth and mimics the effects of NRP1 silencing.
• The NRP1–AGER axis regulates oxidative stress–mediated tumor progression in NSCLC.

## Introduction

Lung cancer remains the leading cause of cancer-related mortality worldwide, with its incidence markedly increasing in the elderly population. The frequency of diagnosis in patients over 70-years old is two to three times higher than in younger individuals.^1^ According to the Surveillance, Epidemiology, and End Results (SEER) database, the median age at diagnosis for Non-Small Cell Lung Cancer (NSCLC) in the United States is 71-years.^1^ Given these statistics, evaluating the mechanisms and therapeutic strategies for age-related lung cancer is crucial, especially since lung and cardiovascular functional decline in the elderly often makes radical surgical resection impractical.^2^ Understanding the molecular basis of aging-associated lung cancer may therefore facilitate the development of non-invasive diagnostic and therapeutic approaches for this population.

Neuropilin-1 (NRP1) is a non-tyrosine kinase transmembrane glycoprotein that is highly expressed in multiple tumor types and plays key roles in angiogenesis, proliferation, and metastasis.^3^^,^^4^ Notably, NRP1 expression is elevated in NSCLC tissues,^5^ and HIF-1α can upregulate NRP1 under hypoxic conditions to promote vasculogenic mimicry in lung adenocarcinoma.^6^ However, whether NRP1 participates in the pathogenesis of aging-related lung cancer remains largely unknown. Recent studies indicate that NRP1 localizes to mitochondria and regulates mitochondrial dynamics, with NRP1 silencing leading to mitochondrial dysfunction.^7^ Furthermore, NRP1 overexpression has been shown to induce ROS production,^8^ and accumulating evidence suggests that Reactive Oxygen Species (ROS) accumulation promotes tumor cell apoptosis.^9^ In parallel, bioinformatics analyses have identified a strong correlation between NRP1 and the Advanced Glycation End-product-specific Receptor (AGER/RAGE), whose expression is reduced in lung cancer tissues. Overexpression of AGER suppresses NSCLC malignancy^10^ and induces broad activation of ROS-related signaling pathways.^11^

Based on these findings, the present study aimed to investigate whether NRP1 silencing promotes mitochondrial apoptosis in NSCLC cells through AGER-mediated ROS activation, thereby uncovering a potential molecular mechanism and therapeutic strategy for aging-associated lung cancer.

## Materials and methods

### Data sources

Transcriptomic data were obtained from The Cancer Genome Atlas (TCGA) and the Gene Expression Omnibus (GEO, https://www.ncbi.nlm.nih.gov/geo) databases, including the datasets TCGA-LUAD (522 tumor and 59 normal samples), TCGA-LUSC (493 tumor and 51 normal samples), GSE19188 (91 tumor and 65 normal samples), GSE30219 (293 tumor and 14 normal samples), and GSE116959 (57 tumor and 11 normal samples). Probe IDs were converted to gene symbols according to platform annotation files. Probes corresponding to multiple genes were excluded, while for genes represented by multiple probes, the mean expression value was used. Prior to downstream analyses, potential batch effects resulting from dataset integration were evaluated and removed using the “ComBat” algorithm from the “sva” R package. Expression data were normalized using the “normalizeBetweenArrays” function in the R package “limma” to ensure comparable expression distributions across arrays.

### Differential expression analysis of NRP1 and lung cancer subtypes

The TCGA-LUAD and TCGA-LUSC datasets were merged to form the TCGA-LUNG cohort. Differentially Expressed Genes (DEGs) were identified using the “limma” package to compare gene expression levels between lung cancer and normal tissues and between NRP1 high and low-expression groups. DEGs were defined as those with |log2FC| > 1 and adjusted p-value < 0.05. Differential expression patterns were visualized using heatmaps generated with pheatmap and volcano plots generated with “ggplot2”.

### Functional enrichment analysis of differentially expressed genes

Kyoto Encyclopedia of Genes and Genomes (KEGG) enrichment analyses were performed using the “clusterProfiler” package in R. KEGG pathway analysis was conducted to determine significantly enriched signaling pathways. Enrichment terms with adjusted p-value <0.05 were considered statistically significant.

### WGCNA for identification of lung cancer gene modules

Lung cancer status and age were used as phenotypic traits for Weighted Gene Co-expression Network Analysis (WGCNA). Genes with variance above the top 25% were retained for network construction. An optimal soft-thresholding power (β = 6) was selected based on the scale-free topology criterion (R-squared > 0.85) using the “pickSoftThreshold” function. A hierarchical clustering tree and dynamic tree-cutting algorithm were applied to identify distinct gene modules. Module-trait relationships were determined based on correlations with age and lung cancer traits. The modules significantly correlated with both factors were selected for further analysis. The core genes from these modules were intersected with the previously identified Differentially Expressed Genes (DEGs) to construct a Venn diagram and obtain the shared candidate genes.

### Identification of feature genes via correlation analysis

Spearman correlation analysis was conducted using the “corrplot” package to evaluate the association between the expression of module genes and both NRP1 expression and age. To strictly control the false discovery rate, all p-values from the Spearman correlation analyses were adjusted using the Benjamini-Hochberg (BH) method. Genes showing significant correlations with both NRP1 and age (BH-adjusted p-value < 0.05) were considered retained for subsequent analyses.

### Screening of common key genes using multiple machine learning algorithms

To minimize potential bias in diagnostic gene selection, three independent machine learning algorithms were applied: Least Absolute Shrinkage and Selection Operator (LASSO) regression, Extreme Gradient Boosting (XGBoost), and Random Forest (RF). LASSO regression was performed using the glmnet package with 10-fold cross-validation. XGBoost analysis was conducted using the caret package, and feature importance was ranked based on the Gain metric. Random Forest analysis was implemented via the “randomForest” package, with gene importance evaluated by the Mean Decrease Gini index. Genes identified as important features by both XGBoost and RF were intersected with those selected by LASSO to obtain the final common key genes.

### Validation of the key candidate genes

To ensure the robustness and generalizability of the identified key genes and to mitigate the risk of algorithmic overfitting, three independent external datasets (GSE19188, GSE30219, and GSE116959) were utilized. The expression patterns of the final candidate genes identified by the machine learning pipeline were verified across these external cohorts using Student’s *t*-tests or Wilcoxon rank-sum tests, as appropriate.

### Human tissue samples

Human NSCLC tissues and corresponding adjacent non-tumorous lung tissues were obtained from six patients who underwent surgical resection at Huai’an Second People’s Hospital. To specifically investigate the age-associated expression profiles, the enrolled patients were stratified into two distinct cohorts: an elderly group (≥ 65 years, n = 14) and a younger group (< 65 years, n = 12). None of the patients had received chemotherapy or radiotherapy prior to surgery. All tissue specimens were immediately snap-frozen in liquid nitrogen and stored at −80 °C until further analysis. All procedures involving human samples were conducted in accordance with the Declaration of Helsinki and approved by the Ethics Committee of Huai’an Second People’s Hospital (approval number: HEYLL2025114; approved on November 10, 2025). Written informed consent was obtained from all participants prior to sample collection. Furthermore, the reporting of the clinical and observational components of this study strictly conforms to the STROBE (Strengthening the Reporting of Observational Studies in Epidemiology) guidelines.

### Cell culture and transfection

Human Bronchial Epithelial cells (16HBE) and lung cancer cell lines (A549, NCI-H1299, NCI-H522) were obtained from Procell and Cybercon. Cells were cultured in RPMI-1640 medium (Hyclone) supplemented with 10% Fetal Bovine Serum (FBS), 100 U/mL penicillin, and 100 μg/mL streptomycin at 37 °C in a humidified incubator with 5% CO_2_. Frozen cells were rapidly thawed at 37 °C, centrifuged at 1000 rpm for 5 min, and resuspended in complete medium before seeding. Cells were passaged at ∼80% confluence using 0.25% trypsin-EDTA.

For transient transfection, cells at 70%–80% confluence were transfected with specific small interfering RNAs (siRNAs) or overexpression plasmids using Lipofectamine™ 2000 (Invitrogen) in serum-free Opti-MEM (Gibco) for 4–6 h, followed by replacement with fresh complete medium. The siRNAs included siRNA-NRP1, siRNA-AGER, and a non-targeting scrambled siRNA (siRNA-NC), which served as a negative control. For overexpression studies, an AGER overexpression plasmid or an empty vector backbone (OV-NC) was utilized. The nucleic acid to Lipofectamine ratio was maintained at 1 μg: 2–3 μL. Furthermore, to evaluate the specific role of ROS, cells transfected with siRNA-NRP1 were subsequently treated with the ROS scavenger N-acetyl-L-cysteine (NAC, 5 mM) for 30 min prior to downstream functional assays. Cell growth and morphology were monitored using an inverted microscope (Olympus CKX53/IX53). Transfection efficiency was comprehensively evaluated by quantitative Real-Time PCR (qRT-PCR) and Western blotting.

### RNA extraction and qRT-PCR

Total RNA was extracted using Trizol reagent (Novizan). RNA concentration and purity were measured using a NanoDrop spectrophotometer. cDNA was synthesized from 2 μg RNA using Hifair® AdvanceFast 1st Strand cDNA Synthesis SuperMix (YEASEN) in a 20 μL reaction. qRT-PCR was performed using Hieff UNICON® ColorGPS qPCR SYBR Green Master Mix (YEASEN) on a Roche LightCycler® 480II. Each 20 μL reaction contained 10 μL SYBR Green, 1 μL cDNA, 0.4 μL each primer (10 μM), and 8.2 μL RNase-free water. Cycling conditions: 95 °C for 5 min; 40 cycles of 95 °C for 10 s, 60 °C for 30 s. Relative expression was calculated using the 2^−ΔΔCt^ method, normalized to β-actin. Primer sequences are in Supplementary Table 1.

### Western blot

Cells were lysed in RIPA buffer with 1 mM PMSF, incubated on ice for 30 min, and centrifuged at 12,000 rpm for 20 min at 4 °C. Protein concentrations were determined using a BCA assay kit (Biosharp). Proteins (20*–*30 μg) were separated on 8%*–*12% SDS-PAGE gels prepared from Tris, glycine, SDS, acrylamide, N,N′-methylenebisacrylamide, ammonium persulfate, and TEMED (Guoyao/BIOFROXX), and transferred to PVDF membranes (methanol-activated) using a Tenon EPS-200 apparatus at 300 mA for 2 h. Membranes were blocked with 5% nonfat milk in TBST for 1 h at room temperature, incubated with primary antibodies (AGER, CDK4, CDK6, BCL2, Bax, CytC, NRP1, β-actin; Affinity/Proteintech, 1:1000) overnight at 4 °C, washed, and incubated with HRP-conjugated secondary antibodies (FITC- or Cy3-conjugated goat anti-mouse/rabbit; Servicebio, 1:10,000) for 30 min. Bands were visualized with ECL chemiluminescence (Tenon Tanon5200).

### Co-Immunoprecipitation (Co-IP)

To detect the endogenous interaction between NRP1 and AGER, cells were lysed in NP-40 lysis buffer supplemented with protease inhibitors. Equal amounts of protein lysates were incubated with anti-NRP1, anti-AGER, or control IgG antibodies overnight at 4 °C, followed by incubation with Protein A/G magnetic beads. The beads were then washed, and the immunoprecipitated complexes were boiled in SDS loading buffer and analyzed by Western blotting.

### Cell viability and proliferation assays

Cell viability was measured using the CCK-8 assay (Beyotime). Cells were seeded at 5 × 10^3^ cells/well in 96-well plates, incubated overnight, treated as indicated, and then 10 μL CCK-8 solution was added per well for 1*–*4 h at 37 °C. Absorbance at 450 nm was measured using a microplate reader (Thermo MK3).

Cell proliferation was assessed using BeyoClick™ EdU-488 (Beyotime). Cells were seeded at 1 × 10^5^ cells/well in 6-well plates, incubated with 10 μM EdU for 2 h at 37 °C, fixed with 4% paraformaldehyde for 15 min, permeabilized with 0.3% Triton X-100 for 10 min, incubated with Click reaction solution for 30 min at room temperature in the dark, and counterstained with Hoechst 33,342 (1:1000) for 10 min. Images were captured under an inverted fluorescence microscope (Olympus IX53).

### Flow cytometry

Cell cycle and apoptosis were analyzed by flow cytometry (BD or equivalent). For cell cycle, ethanol-fixed cells were stained with PI/RNase solution (0.5 mL/sample) at 37 °C for 30 min in the dark. For apoptosis, cells were stained with Annexin V-FITC/PI (10 μL/5 μL in 300 μL binding buffer) for 15 min at room temperature in the dark. Samples were filtered through a 400-mesh strainer and analyzed on a BD flow cytometer; data were processed with FlowJo software.

### ROS, mitochondrial membrane potential, and caspase-3 activity assays

Cells were seeded at 5 × 10^5^ cells/well in 12-well plates. ROS generation was measured using DCFH-DA (10 μM) for 30 min at 37 °C in the dark. Mitochondrial membrane potential was assessed using JC-1 dye (0.5 mL/well) for 30 min at 37 °C, washed twice, and caspase-3 activity was measured using SuperView™ 488 Caspase-3 Kit (Beyotime/Yesen/Novizan): cells were incubated with 5 μM substrate for 30 min, washed, and counterstained with Hoechst 33,342 (1:1000) for 10 min. Fluorescence images were captured using an Olympus IX53 microscope. Intracellular ATP levels were quantified using a commercial ATP Assay Kit (HY-U00451, MCE) according to the manufacturer’s instructions. Luminescence was measured using a microplate reader, and the relative ATP levels were calculated by normalizing to the control group.

### ELISA

Cellular MDA and SOD levels were measured using commercial ELISA kits (Mmbio). Samples and standards (50 μL) were added in duplicate to 96-well plates, incubated with 50 μL biotin-labeled antibody at 37 °C for 1 h, washed, and incubated with 80 μL HRP-conjugated streptavidin for 30 min. Substrate solutions (50 μL each) were added for 10 min at 37 °C in the dark, and reactions were terminated with 50 μL stop solution. Absorbance was measured at 450 nm (Thermo MK3).

### Statistical analysis

All statistical analyses in this study were performed using R software (v4.4.0) and GraphPad Prism (v8.0). The differences between the two groups were estimated employing an independent Student *t*-test and Mann-Whitney *U test*. If not specifically indicated, correlation coefficients between different molecules were calculated utilizing Spearman’s correlation analysis throughout this study. All statistical tests were two-sided, and P lower than 0.05 was indicative of statistical significance.

## Results

### Identification of NRP1– and lung cancer-related genes

**Fig. 1:**
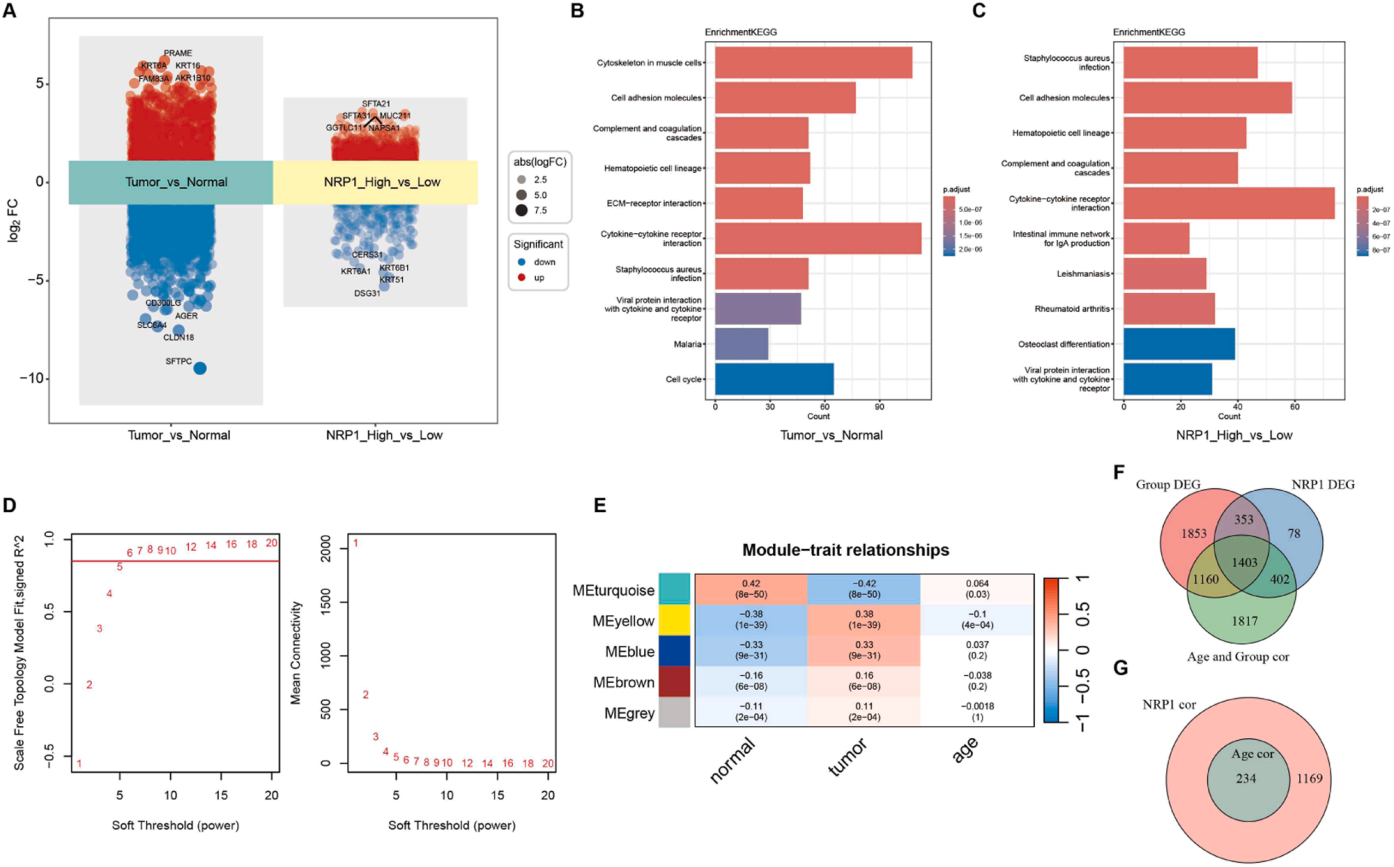
**Identification of NRP1-related genes in the training set.** (A) Differentially expressed genes between TCGA-LUNG cancer and control samples (left) and TCGA-LUNG NRP1 high and low samples (right). (B–C) KEGG enrichment analysis results for differentially expressed genes between TCGA-LUNG cancer and control samples (B) and TCGA-LUNG NRP1 high and low samples (C). (D) Determination of soft-thresholding power β=6 based on scale-free topology. (E) Heatmap of the correlation between the module and lung cancer and age. (F) Intersection Venn diagram of differentially expressed genes between NRP1 high and low groups, differentially expressed genes between lung cancer and control groups, and module genes associated with age and lung cancer. (G) Intersection Venn diagram of 1403 genes significantly associated with NRP1 and age in univariate correlation analysis.

Differential expression analysis identified a total of 4769 DEGs between tumor and normal samples in the TCGA-LUNG cohort, including 2690 downregulated and 2079 upregulated genes (Fig. 1A). Similarly, comparison between NRP1 high- and low-expression groups revealed 2236 DEGs, of which 374 were downregulated and 1862 were upregulated (Fig. 1A). KEGG pathway enrichment analysis demonstrated that the DEGs from both comparisons were significantly associated with pathways such as cell cycle, cell adhesion molecules, and cytokine-cytokine receptor interaction, suggesting that NRP1 may influence these biological processes and play a functional role in lung cancer development (Fig. 1B–C). To further identify genes correlated with both lung cancer and age, the authors selected the top 10,000 genes with the highest median absolute deviation from the preprocessed TCGA-LUNG expression matrix. A soft-thresholding power of β=6 was chosen to construct a scale-free network (Fig. 1D). Based on average linkage hierarchical clustering and dynamic tree cutting, five co-expression modules were identified (Fig. 1E). Among these, the turquoise module showed the strongest negative correlation with lung cancer and a positive correlation with age, and was therefore defined as the age-lung cancer-related module. This module contained 4782 genes, which were considered age-associated lung cancer genes (Fig. 1E). The intersection of NRP1 high/low DEGs, lung cancer vs. normal DEGs, and age-related module genes yielded 1403 overlapping genes that may be jointly related to NRP1, age, and lung cancer (Fig. 1F). Further univariate correlation analysis showed that 1169 genes were significantly correlated with NRP1 expression, whereas only 234 genes were significantly correlated with age. These 234 genes were therefore defined as NRP1- and age-associated lung cancer genes (Fig. 1G).

### Machine learning-based screening of key feature genes

**Fig. 2:**
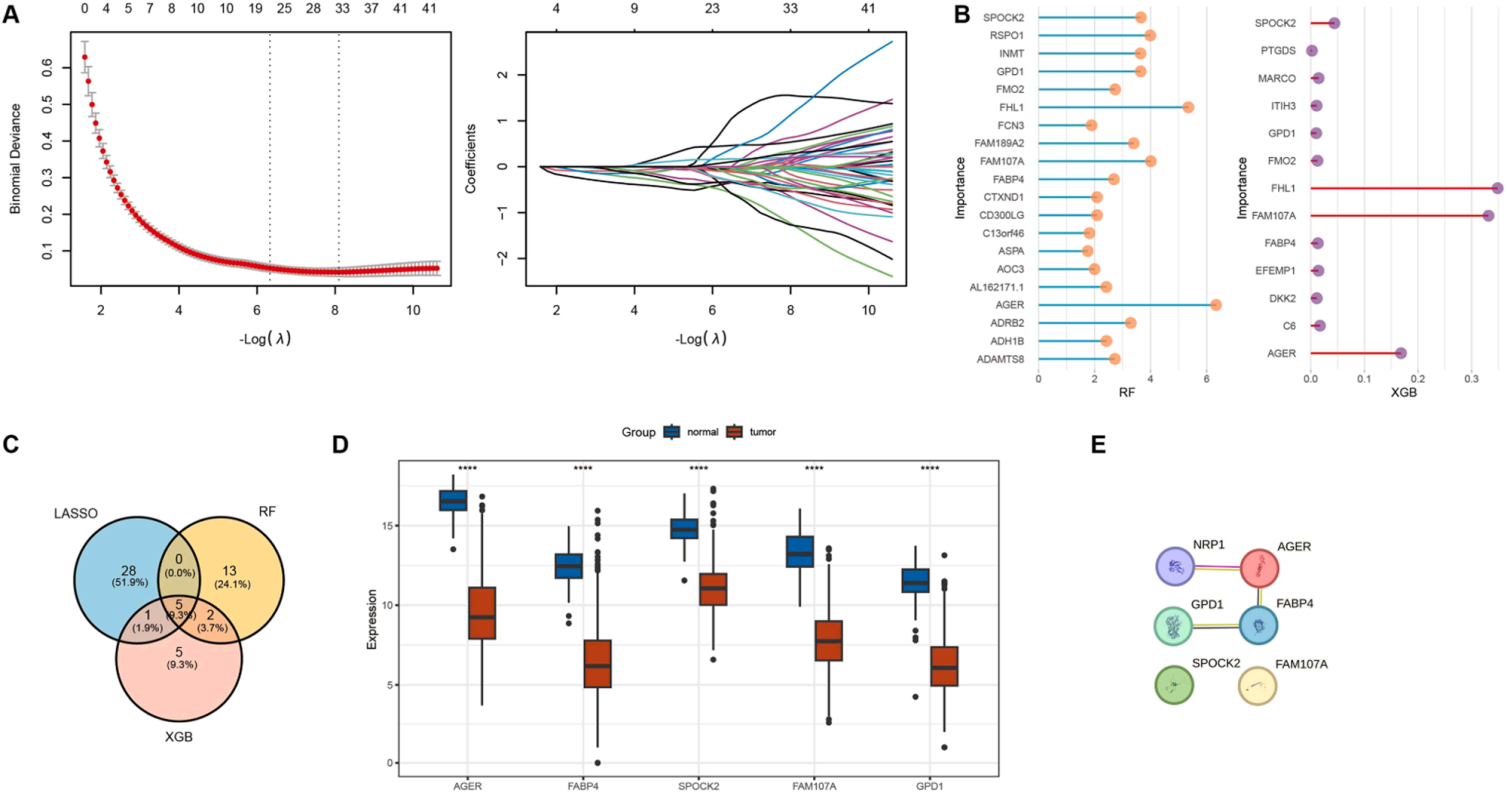
**Feature screening and expression validation by machine learning.** (A) Feature gene screening by LASSO regression analysis. (B) Analysis of key feature genes using RF (left) and XGBoost (right). (C) Intersection Venn diagram of key genes selected by three machine learning methods. (D) Expression of five key genes in the TCGA-LUNG cancer and control samples. Blue represents normal and red represents tumor. (E) PPI network of the five key genes and NRP1.

The 234 candidate genes were further analyzed using machine learning algorithms to identify key diagnostic biomarkers. In LASSO regression, model performance was optimal when 34 genes were included (Fig. 2A). In parallel, RF and XGBoost analyses identified the top 20 and top 13 genes with the highest feature importance, respectively, with 7 overlapping genes between the two methods (Fig. 2B). Taking the intersection of key genes identified by all three algorithms, five genes – AGER, SPOCK2, FABP4, FAM107A, and GPD1 – were consistently selected as the most candidate biomarkers (Fig. 2C). Validation across four independent datasets (TCGA-LUNG, GSE19188, GSE30219, and GSE116959) confirmed that these five genes were consistently downregulated in lung cancer tissues compared with normal tissues (Fig. 2D; Supplementary Fig. 1). The consistent expression patterns across datasets supported the reliability and universality of these key genes in lung cancer. Moreover, STRING database analysis revealed potential interactions between NRP1 and AGER, suggesting that AGER may directly interact with NRP1 (Fig. 2E).

### Expression of NRP1 and AGER in NSCLC tissues and cell lines

**Fig. 3:**
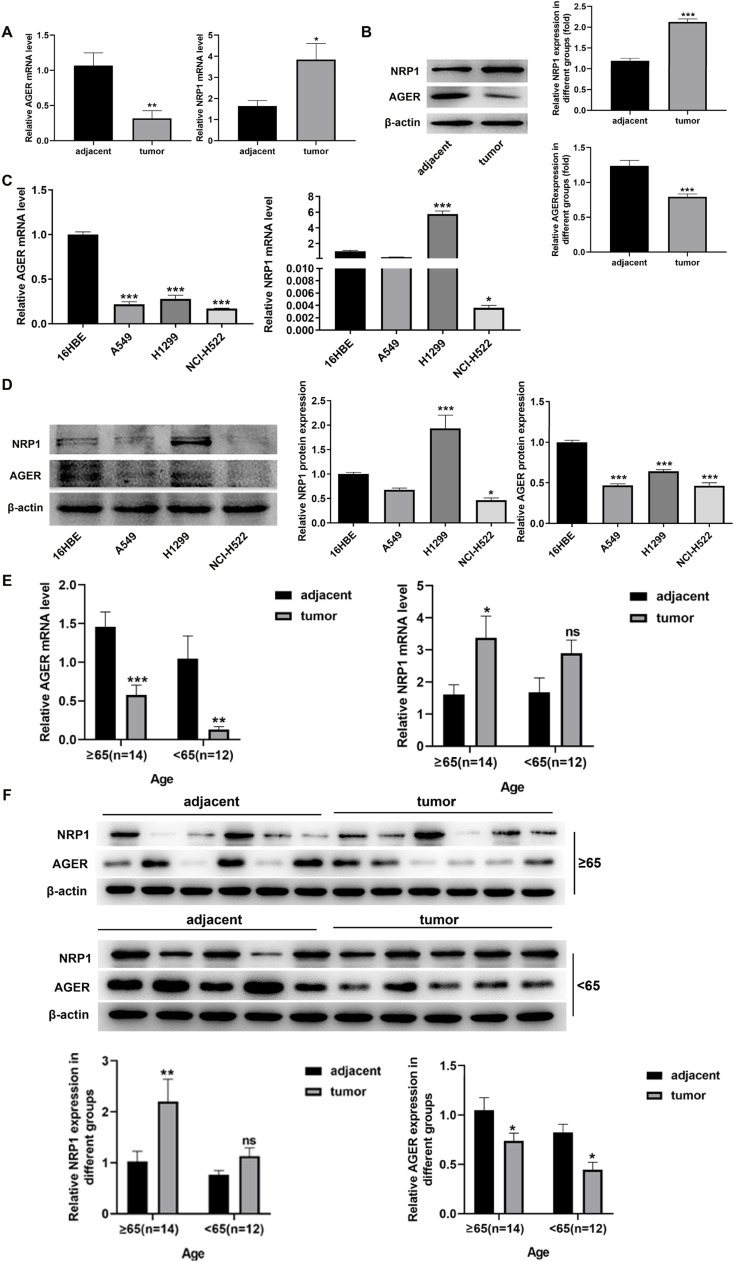
Expression of NRP1 and AGER in NSCLC tissues and cell lines. (A–B) RT-qPCR (A) and Western blot (B) analysis of NRP1 and AGER expression in normal lung and NSCLC tissues (n = 6). (C–D) RT-qPCR (C) and Western blot (D) analysis of NRP1 and AGER expression in different cell lines (16HBE, H1299, A549, and NCI-H522) (n = 3). (E–F) qPCR and Western blotting analysis of NRP1 and AGER expression levels in NSCLC tissues and adjacent normal tissues, stratified by patient age (≥ 65-years, n = 14; < 65-years, n = 12). Data are presented as the mean ± SD of three independent experiments. * p < 0.05, ** p < 0.01, *** p < 0.001.

RT-qPCR and Western blot analyses revealed that, compared with normal lung tissues, NRP1 expression was significantly upregulated, whereas AGER expression was markedly downregulated in NSCLC tissues (Fig. 3A–B). In cultured cells, RT-qPCR and Western blot results demonstrated that, compared with the normal bronchial epithelial cell line 16HBE, NRP1 expression was significantly increased, and AGER expression was decreased in H1299 cells. In contrast, both NRP1 and AGER expression levels were reduced in A549 and NCI-H522 cells (Fig. 3C–D). Although differential expression patterns of NRP1 and AGER were observed among NSCLC cell lines, H1299 cells displayed the expression profile most consistent with that of clinical NSCLC tissues, characterized by elevated NRP1 and reduced AGER expression. Given that NSCLC exhibits substantial molecular heterogeneity across different cell lines, H1299 cells were selected as an appropriate in vitro model for subsequent mechanistic investigations.

To experimentally validate the age-dependency of the NRP1-AGER axis inferred from the WGCNA analysis, the authors expanded the present clinical cohort and stratified the collected NSCLC and matched adjacent normal tissues into an elderly group (≥65-years, n = 14) and a younger group (< 65-years, n = 12). RT-qPCR and Western Blot analyses revealed a striking age-specific expression pattern. Specifically, NRP1 expression was significantly upregulated in tumor tissues compared with adjacent normal tissues exclusively in the elderly group, whereas this upregulation was not statistically significant in the younger cohort (Fig. 3E). Conversely, AGER expression was significantly downregulated in tumor tissues across both age groups (Fig. 3F).

### Silencing of NRP1 modulates cell proliferation and induces cell cycle arrest in NSCLC cells

**Fig. 4:**
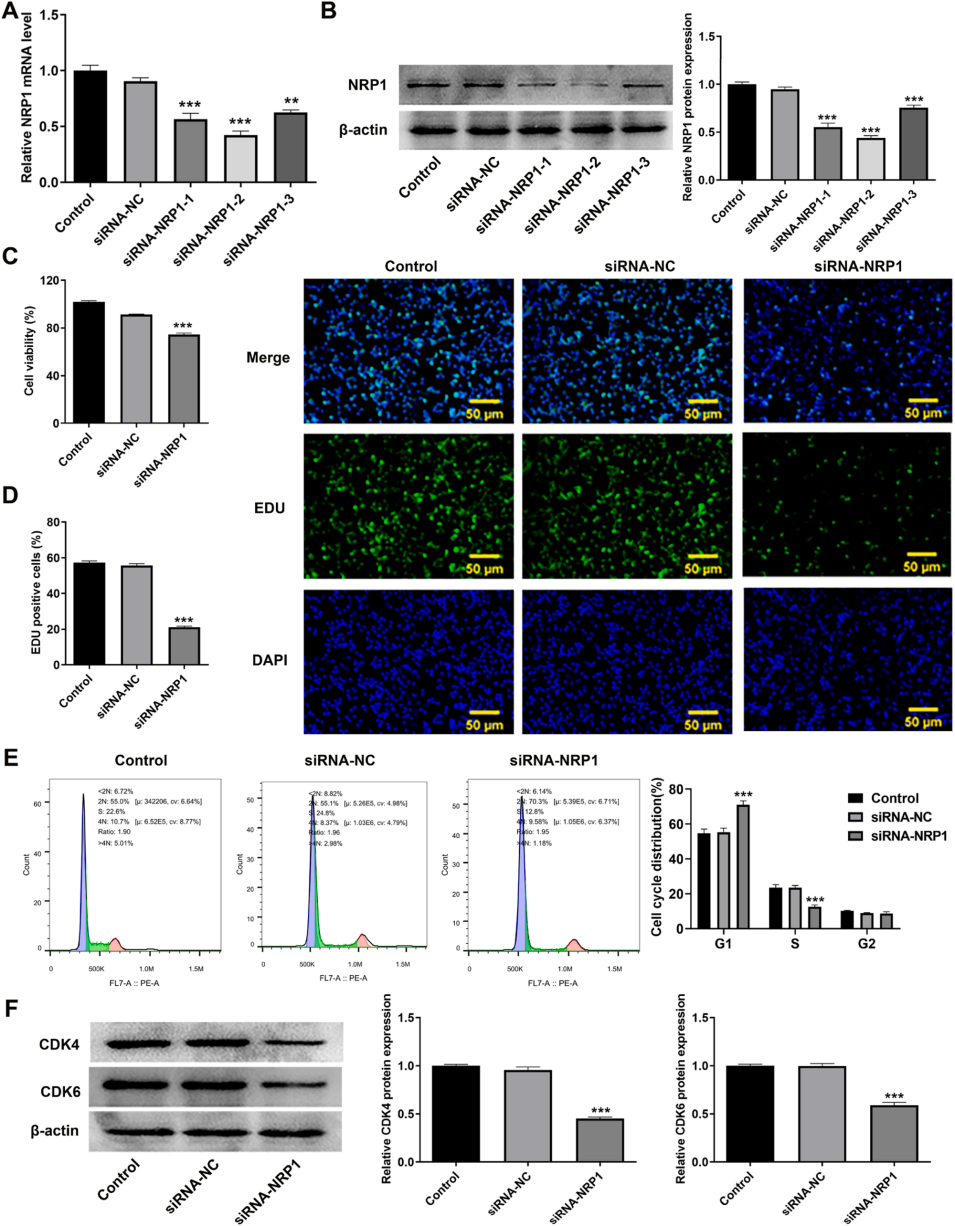
**Silencing of NRP1 inhibits proliferation and induces G1 phase arrest in NSCLC cells.** (A–B) RT-qPCR (A) and Western blot (B) analysis of the efficiency of NRP1 knockdown in cells after transfection with siRNA-NRP1. Magnification: 200×. (C–D) CCK-8 (C) and EdU (D) were used to detect cell viability and proliferation rate. Magnification: 200×. (E) Flow cytometry was used to analyze the proportion of cells in the G1 phase and the proportion of cells in the S phase after NRP1 knockdown. (F) Western blot analysis of CDK4 and CDK6 expression after NRP1 silencing. Data are presented as the mean ± SD of three independent experiments (n = 3). * p < 0.05, ** p < 0.01, *** p < 0.001.

To explore the functional role of NRP1 in NSCLC, an NRP1 interference plasmid was constructed and transfected into H1299 cells. RT-qPCR and Western blot analyses confirmed that NRP1 expression was markedly reduced in all three siRNA-NRP1 groups (siRNA-NRP1– 1, −2, and −3) compared with the siRNA-NC group, with siRNA-NRP1– 2 showing the most potent silencing effect (Fig. 4A–B). Thus, siRNA-NRP1– 2 was used for subsequent studies. CCK-8 and EdU assays revealed that NRP1 silencing significantly reduced cell viability and proliferation rates compared with the control group (Fig. 4C–D). Flow cytometry analysis indicated that the proportion of cells in the G1 phase was markedly increased, whereas that in the S phase was decreased following NRP1 knockdown (Fig. 4E). Moreover, the expression of cell cycle–related proteins CDK4 and CDK6 was significantly decreased (Fig. 4F). Collectively, these data suggest that NRP1 knockdown inhibits NSCLC cell proliferation and induces G1 phase arrest.

### NRP1 knockdown promotes mitochondria-dependent apoptosis in NSCLC cells

**Fig. 5:**
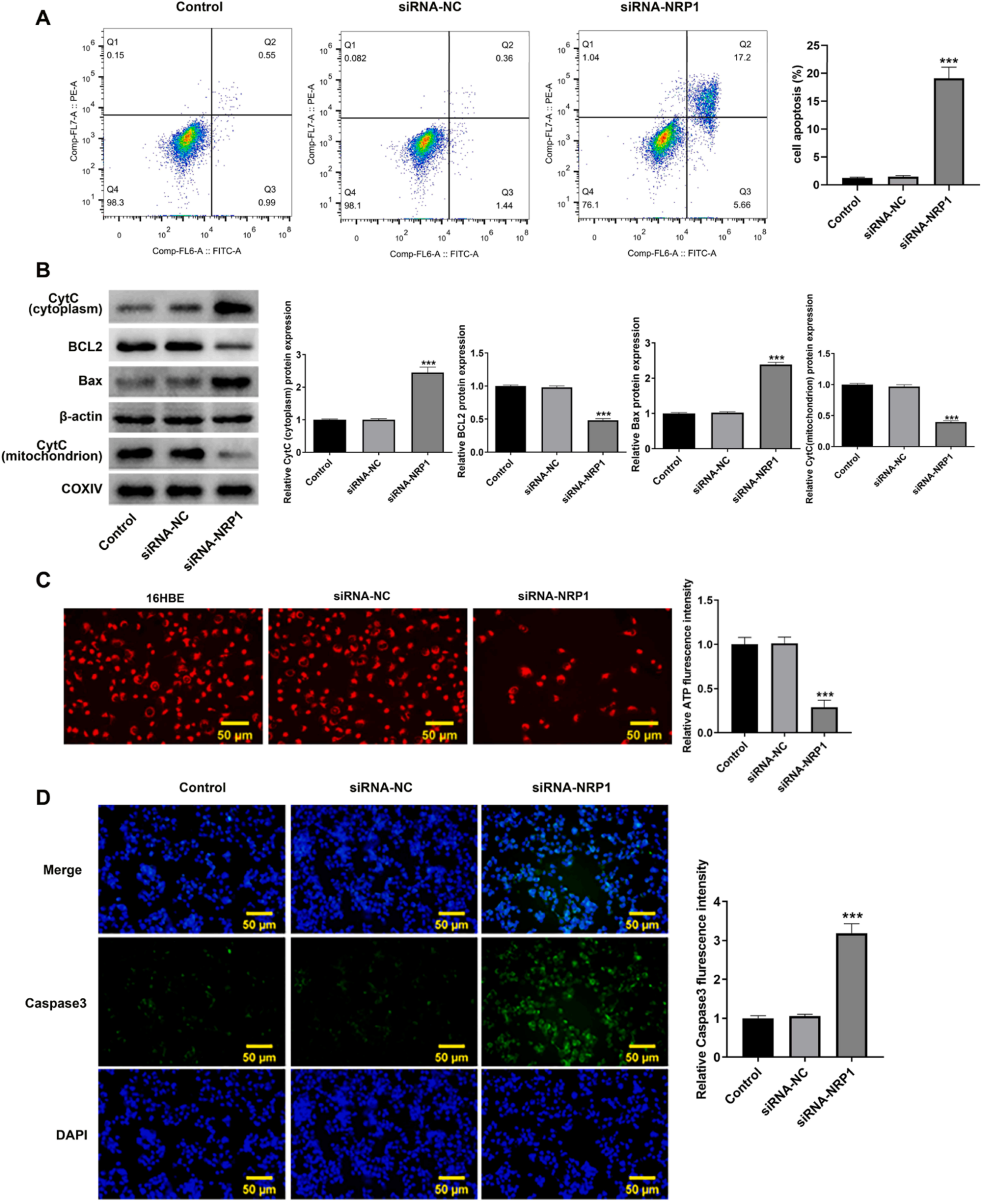
**NRP1 knockdown promotes mitochondria-dependent apoptosis in NSCLC cells.** (A) Flow cytometry was used to detect the level of cell apoptosis. (B) Western blot was used to detect the expression of apoptosis-related proteins CytC (cytoplasm, mitochondria), BCL2, and Bax. (C) Detection of caspase-3 activity level. Magnification: 200×. D ATP kit for detecting mitochondrial function in different groups of H1299 cells. Magnification: 200×. Data are presented as the mean ± SD of three independent experiments (n = 3). * p < 0.05, ** p < 0.01, *** p < 0.001.

To further investigate whether NRP1 knockdown induces apoptosis, flow cytometric analysis was performed. The results showed a marked increase in apoptotic cells in the siRNA-NRP1 group compared with the siRNA-NC group (Fig. 5A). Western blot analysis revealed that Cytosolic Cytochrome c (CytC) and Bax levels were significantly elevated, while mitochondrial CytC and BCL2 levels were markedly decreased following NRP1 knockdown (Fig. 5B–C). To further validate mitochondrial dysfunction functionally, intracellular ATP levels were assessed. Consistent with the loss of mitochondrial membrane potential, H1299 cells transfected with siRNA-NRP1 exhibited a severe reduction in ATP production compared to the control group (Fig. 5D). Consistently, Caspase-3 activity was significantly enhanced in siRNA-NRP1–transfected cells compared with controls (Fig. 5E). These findings demonstrate that NRP1 silencing triggers mitochondrial dysfunction and promotes mitochondria-dependent apoptosis in NSCLC cells.

### NRP1 silencing enhances oxidative stress in NSCLC cells

To examine the potential relationship between NRP1 and Reactive Oxygen Species (ROS), DCFH-DA staining and ELISA assays were performed. The results indicated that NRP1 knockdown markedly elevated ROS levels, increased MDA, decreased SOD, and caused a loss of mitochondrial membrane potential in H1299 cells (Fig. S2). These preliminary results suggest that NRP1 silencing induces oxidative stress in NSCLC cells, prompting us to further investigate the downstream mediators.

### NRP1 knockdown has no significant effect on normal bronchial epithelial cells

To further evaluate whether the biological effects of NRP1 silencing are specific to NSCLC cells, additional experiments were performed in the normal human bronchial epithelial cell line 16HBE. EdU staining demonstrated that NRP1 knockdown did not significantly affect the proliferative capacity of 16HBE cells compared with the siRNA-NC group (Fig. S3). Consistently, flow cytometric analysis revealed no significant difference in apoptosis following NRP1 silencing in 16HBE cells. Moreover, intracellular ROS levels remained largely unchanged after NRP1 knockdown. These findings suggest that the effects of NRP1 depletion on oxidative stress, apoptosis, and proliferation are more prominent in NSCLC cells than in normal bronchial epithelial cells.

### AGER overexpression inhibits cell proliferation and induces cell cycle arrest in NSCLC cells

**Fig. 6:**
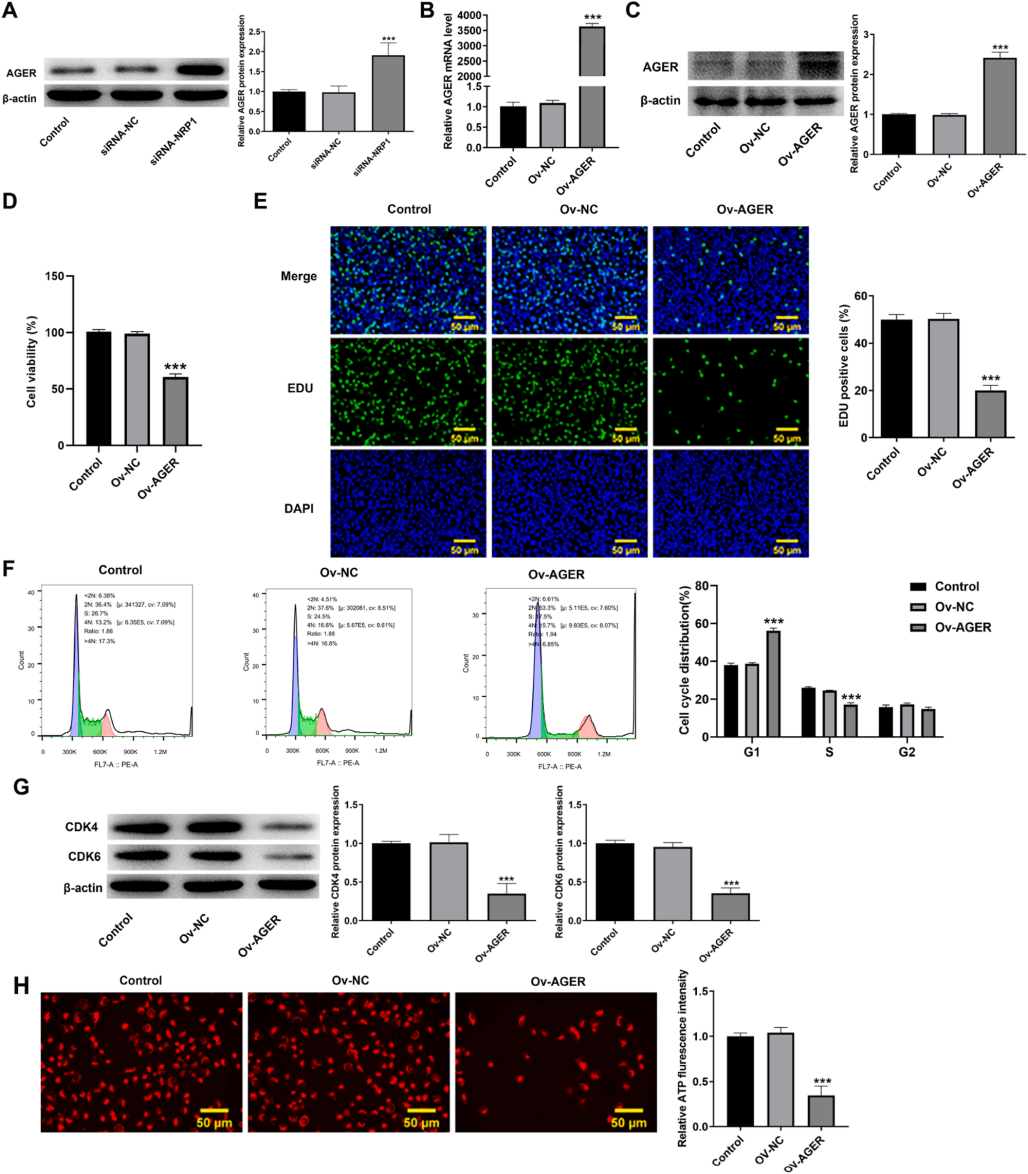
**AGER overexpression inhibits proliferation and induces cell cycle arrest in NSCLC cells.** (A) Western blot analysis AGER expression in H1299 cells. (B–C) Construct an AGER overexpression plasmid, and detect the AGER overexpression level by RT-qPCR and western blot. (D–E) CCK-8 (D) and EdU (E) were used to detect cell viability and proliferation rate. Magnification: 200×. (F) Flow cytometry was used to analyze the proportion of cells in the G1 phase and the proportion of cells in the S phase after AGER overexpression. (G) Western blot analysis of CDK4 and CDK6 expression after NRP1 silencing. (H) ATP kit for detecting mitochondrial function in different groups of H1299 cells. Magnification: 200×. Data are presented as the mean ± SD of three independent experiments (n = 3). OV-NC, cells transfected with empty vector plasmid. * p < 0.05, ** p < 0.01, *** p < 0.001.

Given the inverse expression pattern of AGER, the authors next investigated whether AGER plays a similar inhibitory role in NSCLC progression. Western blot analysis confirmed that AGER expression was significantly enhanced following NRP1 knockdown (Fig. 6A). To further elucidate its function, an AGER overexpression plasmid was transfected into H1299 cells (Fig. 6B–C). CCK-8 and EdU assays showed that AGER overexpression significantly decreased cell viability and proliferation compared with the OV-NC group (Fig. 6D–E). Flow cytometry analysis revealed a substantial increase in the G1 phase population and a decrease in the S phase population in AGER-overexpressing cells (Fig. 6F). Furthermore, Western blot results demonstrated that CDK4 and CDK6 expression levels were markedly reduced in the OV-AGER group relative to controls (Fig. 6G). Additionally, functionally consistent with the effects of NRP1 silencing, cells transfected with the AGER overexpression plasmid exhibited a severe reduction in intracellular ATP levels compared to the OV-NC group (Fig. 6H). Taken together, these results indicate that AGER overexpression modulates NSCLC cell proliferation and induces G1 phase arrest, suggesting that AGER may act as a downstream effector of NRP1 in regulating tumor growth.

### NRP1 interacts with AGER to regulate ROS-dependent mitochondrial apoptosis in NSCLC cells

**Fig. 7:**
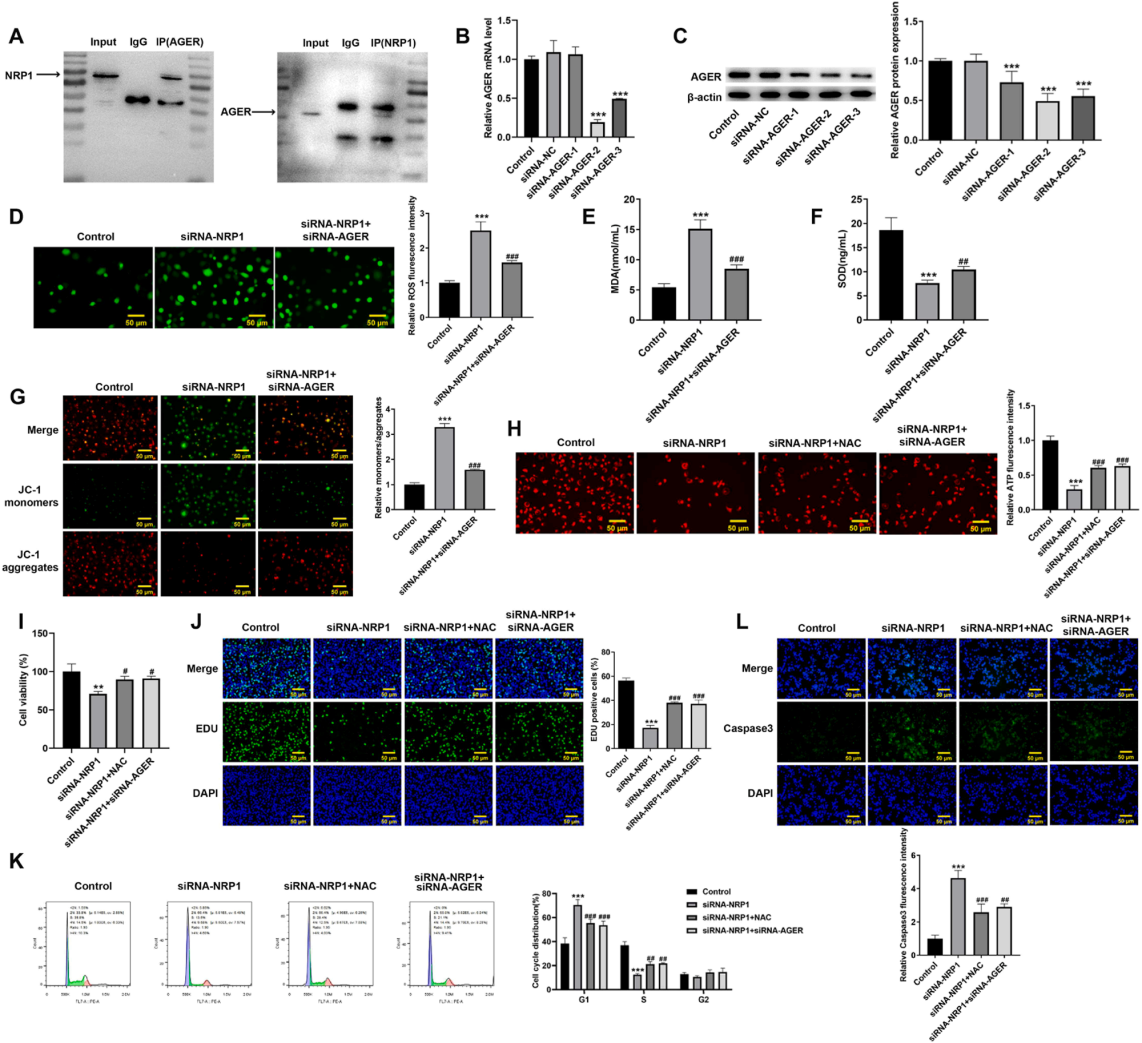
**NRP1 interacts with AGER to regulate ROS-dependent mitochondrial apoptosis and proliferation in NSCLC cells.** (A) Co-Immunoprecipitation (Co-IP) assay demonstrating the endogenous physical interaction between NRP1 and AGER proteins in H1299 cells. (B–C) Validation of AGER knockdown efficiency in H1299 cells transfected with siRNA-AGER-2 or siRNA-AGER-3 compared to the negative control (siRNA-NC). siRNA-AGER-2 was selected for subsequent rescue experiments due to its optimal silencing effect. (D) Intracellular ROS levels were evaluated using DCFH-DA staining in H1299 cells treated with siRNA-NC, siRNA-NRP1, siRNA-NRP1 + siRNA-AGER-2, or siRNA-NRP1 + NAC (5 mM). Magnification: 200×. (E–F) The concentrations of MDA (E) and the activity of SOD (F) in the indicated groups were measured by ELISA assays to assess lipid peroxidation and antioxidant capacity. (G) Mitochondrial membrane potential was analyzed by JC-1 staining. An increased ratio of JC-1 monomers (green fluorescence) to aggregates (red fluorescence) indicates a loss of mitochondrial membrane potential. Magnification: 200×. (H) ATP kit for detecting mitochondrial function in different groups of H1299 cells. Magnification: 200×. (I–J) Cell viability and proliferation were assessed using CCK-8 and EdU incorporation assays, respectively, across the designated treatment groups. Magnification: 200×. (K) Flow cytometry analysis of cell cycle distribution, highlighting the reversal of G1-phase arrest by AGER knockdown or NAC treatment. (L) Apoptosis was evaluated by measuring relative Caspase-3 activity using a commercial assay kit. Magnification: 200×. Data are presented as the mean ± SD of three independent experiments (n = 3). p < 0.001 vs. the siRNA-NC or Control group; ##p < 0.01, ###p < 0.001 vs. the siRNA-NRP1 group. NAC, N-Acetyl-L-Cysteine.

To further elucidate the mechanistic relationship between NRP1 and AGER, the authors performed endogenous Co-Immunoprecipitation (Co-IP) assays in H1299 cells. The results demonstrated a direct physical interaction between NRP1 and AGER (Fig. 7A).

To definitively determine whether AGER and downstream oxidative stress are responsible for the suppressive effects of NRP1 silencing, comprehensive rescue experiments were conducted. To effectively silence AGER, multiple specific siRNAs were transfected into H1299 cells. Expression analysis confirmed that both siRNA-AGER-2 and siRNA-AGER-3 significantly reduced AGER expression compared with the siRNA-NC group. Because siRNA-AGER-2 exhibited the most potent knockdown efficiency, it was utilized for all subsequent rescue assays (Fig. 7B–C). First, the authors evaluated oxidative stress and mitochondrial function. Consistent with the authors’ previous findings, NRP1 knockdown significantly increased intracellular ROS levels, elevated MDA production, and decreased SOD activity. Notably, co-transfection with siRNA-AGER-2 dramatically reversed these effects, restoring ROS, MDA, and SOD to near-basal levels (Fig. 7D–F). Furthermore, JC-1 staining showed that the increased monomer-to-aggregate ratio (indicative of mitochondrial membrane potential loss) induced by NRP1 silencing was significantly attenuated by concurrent AGER knockdown (Fig. 7G). Correspondingly, the profound intracellular ATP depletion triggered by NRP1 silencing was effectively rescued upon co-treatment with either siRNA-AGER-2 or the ROS scavenger NAC (Fig. 7H).

Next, the authors assessed cellular phenotypes. The profound reduction in cell viability and proliferation caused by siRNA-NRP1 was significantly rescued by both siRNA-AGER-2 and NAC treatments (Fig. 7I–J). Flow cytometry analysis revealed that the G1-phase arrest induced by NRP1 silencing was also significantly reversed upon co-treatment with either siRNA-AGER-2 or NAC (Fig. 7K). Finally, the pronounced elevation in Caspase-3 activity triggered by NRP1 depletion was significantly abrogated in both the siRNA-NRP1 + siRNA-AGER-2 and siRNA-NRP1 + NAC groups (Fig. 7L). Collectively, these findings demonstrate that AGER acts as a critical functional downstream mediator of NRP1, and that the NRP1-AGER axis regulates NSCLC cell survival and mitochondrial apoptosis via the ROS signaling pathway.

## Discussion

Lung cancer remains one of the leading causes of cancer-related mortality worldwide, and NSCLC accounts for approximately 85% of all lung cancer cases.^12^ Despite advances in surgical techniques and targeted therapies, the prognosis of patients with advanced NSCLC remains unsatisfactory due to tumor recurrence and therapeutic resistance. Therefore, identifying novel molecular regulators involved in NSCLC progression is of great importance.

NRP1, a non-tyrosine kinase transmembrane receptor initially identified as a co-receptor for vascular endothelial growth factor, has been implicated in multiple oncogenic processes, including angiogenesis, proliferation, and metastasis.^13^^,^^14^ Aberrant NRP1 expression has been observed in various cancers such as glioma,^15^ colorectal cancer,^16^ and hepatocellular carcinoma,^17^ where it promotes tumor aggressiveness and therapy resistance. Inhibition of NRP1 expression modulates tumorigenesis in bladder cancer,^18^ while NRP1 knockdown reduces invasion and migration in rhabdomyosarcoma cells.^19^ Consistent with these reports, the present data demonstrated that NRP1 was highly expressed in NSCLC tissues and cells. Functional assays showed that NRP1 knockdown suppressed proliferation and induced cell cycle arrest, accompanied by downregulation of CDK4 and CDK6, two essential regulators of the G1/S transition. These results suggest that NRP1 contributes to NSCLC cell cycle progression and proliferation. Mechanistically, NRP1 has been shown to activate several signaling pathways, including PI3K/Akt,^20^ and TGF-β/Smad,^21^ which collectively promote tumor cell survival. In the present study, silencing NRP1 also led to increased ROS accumulation, mitochondrial membrane potential loss, and caspase-3 activation, indicating that NRP1 may protect tumor cells from oxidative stress-induced mitochondrial apoptosis. These findings extend previous observations by highlighting a potential link between NRP1 and mitochondrial homeostasis in NSCLC cells.

Mitochondria play a central role in regulating cellular metabolism, redox balance, and apoptosis.^22^ Disruption of mitochondrial integrity often leads to the release of pro-apoptotic factors such as cytochrome c and activation of downstream caspases.^23^ In the present study, NRP1 knockdown increased cytosolic CytC and Bax levels while reducing mitochondrial CytC and BCL2 expression, consistent with mitochondrial outer membrane permeabilization and activation of the intrinsic apoptotic pathway.^23^ In parallel, JC-1 staining revealed a marked decline in mitochondrial membrane potential, accompanied by a profound depletion of intracellular ATP, further confirming mitochondrial functional and structural dysfunction. The elevated ROS and MDA levels along with decreased SOD activity observed following NRP1 silencing indicate enhanced oxidative stress, lipid peroxidation, and a compromised antioxidant defense system. Taken together, these results suggest that NRP1 supports tumor cell survival by maintaining mitochondrial stability and redox homeostasis, whereas its depletion disrupts these processes, leading to apoptosis. Interestingly, several studies have reported that NRP1 overexpression confers resistance to oxidative damage and enhances tumor cell viability under stress conditions. For example, NRP1-mediated activation of the PI3K/Akt pathway has been linked to increased antioxidant capacity in breast cancers.^24^ The present findings align with this concept, suggesting that NRP1 functions as a key regulator of oxidative stress tolerance in NSCLC cells. Importantly, NRP1 silencing exerted minimal effects on proliferation, apoptosis, and ROS accumulation in normal bronchial epithelial 16HBE cells, suggesting that NSCLC cells may exhibit increased dependence on NRP1 signaling for maintaining redox homeostasis and survival. These findings further support the tumor-specific role of NRP1 in NSCLC progression.

The turquoise module identified by WGCNA exhibited a positive correlation with age. Crucially, this bioinformatic inference was robustly validated by the age-stratified clinical data, which demonstrated that significant NRP1 upregulation is highly specific to elderly (≥65-years) NSCLC patients. This age-dependent divergence highlights the specific pathological relevance of NRP1 in aging-associated NSCLC. AGER (advanced glycosylation end-product-specific receptor), also known as RAGE, is a member of the immunoglobulin superfamily that serves as a receptor for Advanced Glycation End-products (AGEs) and plays a central role in oxidative stress, aging, inflammation, and cellular stress responses.^25^ Depending on cellular context, AGER can act as either a tumor promoter or suppressor. In both NSCLC tissues and H1299 cells, AGER is lowly expressed, and overexpression can inhibit the malignant behavior of non-small cell lung cancer.^10^ In agreement with these observations, the present data demonstrated that AGER overexpression inhibited NSCLC cell proliferation and induced G1 phase arrest, accompanied by a reduction in CDK4 and CDK6 expression. These findings indicate that AGER negatively regulates cell cycle progression in NSCLC cells. Notably, the observed downregulation of AGERs and upregulation of NRP1 in NSCLC may reflect age-related dysregulation of redox homeostasis, and NRP1 knockdown resulted in increased AGER expression, suggesting a potential regulatory relationship between these two molecules. Importantly, the present rescue experiments further demonstrated that AGER knockdown or ROS scavenging by NAC effectively reversed the inhibitory effects induced by NRP1 silencing, including oxidative stress accumulation, mitochondrial dysfunction, cell cycle arrest, and apoptosis. These findings provide functional evidence that AGER-mediated ROS signaling serves as a critical downstream pathway through which NRP1 regulates NSCLC progression. Moreover, Co-IP assays revealed an association between NRP1 and AGER in NSCLC cells, further supporting the existence of a functional interaction between these two proteins.

Several limitations of this study should be acknowledged. First, although the present bioinformatic inferences were successfully validated in vitro and in a local clinical cohort, the functional conclusions currently lack in vivo confirmation. Future studies utilizing tumor xenograft or genetically engineered mouse models are essential to fully substantiate the physiological relevance of the NRP1-AGER-ROS axis within the complex tumor microenvironment. Second, while the authors experimentally confirmed the age-stratified expression of this axis, the sample size of the local clinical cohort is relatively small; thus, larger, multicenter clinical studies are needed. Third, the varying baseline expression of NRP1 and AGER across different NSCLC cell lines underscores the high molecular heterogeneity of the disease. While the present study strictly focused on the NRP1-high subtype, exploring how alternative oncogenic drivers (such as KRAS mutations in A549 cells) bypass or crosstalk with this axis warrants future investigation. Finally, although the present Co-IP and comprehensive rescue assays established a direct interaction and identified AGER as a downstream functional effector of NRP1, it remains to be determined whether a reciprocal feedback loop exists. Exploring whether alterations in AGER levels reciprocally modulate NRP1 stability or expression will provide a more comprehensive understanding of this dynamic regulatory network.

## Conclusion

In this study, the authors revealed that NRP1 expression was significantly upregulated, whereas AGER expression was markedly downregulated in both NSCLC tissues and cell lines. Functionally, silencing NRP1 inhibited NSCLC cell proliferation, induced G1 phase arrest, promoted mitochondrial apoptosis, and enhanced oxidative stress. Mechanistically, the authors confirmed a direct physical interaction between NRP1 and AGER, and demonstrated that AGER knockdown or ROS scavenging fully rescued the anti-tumor effects of NRP1 silencing. Collectively, these findings indicate that NRP1 and AGER play opposite roles in NSCLC progression and that their interplay may be critical in regulating cell survival and apoptosis.

## Ethics approval and consent to participate

This study was conducted in accordance with the Declaration of Helsinki and approved by the Ethics Committee of Huai’an Second People’s Hospital (approval number: HEYLL2025114, approved on November 10, 2025). Written informed consent was obtained from all participants prior to inclusion in the study.

## Consent for publication

Written informed consent for publication of the data was obtained from all participants.

## Data availability

The transcriptomic datasets analyzed during the current study are available in the TCGA (https://portal.gdc.cancer.gov/) and GEO (https://www.ncbi.nlm.nih.gov/geo/) repositories. The datasets generated and analyzed concerning the local clinical human tissue samples are not publicly available due to the strict protection of patient privacy and confidentiality, but anonymized data can be obtained from the corresponding author upon reasonable request.

## Compliance with reporting guidelines

The design, conduct, and reporting of this study adhere to the relevant established guidelines. Specifically, the collection, analysis, and reporting of the human clinical tissue cohorts conform to the STROBE (Strengthening the Reporting of Observational Studies in Epidemiology) Statement guidelines.

## Funding

This work was supported by the Huai’an Science and Technology Project (Grant n° HAB2024017).

## CRediT authorship contribution statement

**Ya Yang:** Investigation, Methodology, Formal analysis, Visualization, Writing – original draft. **Ying Li:** Formal analysis, Data curation, Writing – review & editing. **Yongjuan Zhao:** Resources. **Wenwen Feng:** Resources. **Pengcheng Yu:** Resources. **Shan Li:** Resources. **Zongli Ding:** Conceptualization, Methodology, Supervision, Project administration, Writing – review & editing.

## Declaration of competing interest

The authors have no relevant financial or non-financial interests to disclose.

## Appendix Supplementary materials

Image, application 1
