# Supplementary material for: NRP1 silencing induces ROS-mediated cell cycle arrest and mitochondrial apoptosis in non-small cell lung cancer via upregulation of AGER
Source: Clinics (Sao Paulo). 2026 Aug 13;81:101080. doi: 10.1016/j.clinsp.2026.101080 (PMC13486836; doi:10.1016/j.clinsp.2026.101080)
Supplement: Supplementary file 1 [file mmc1.docx]

**CLINICS-D-26-00019_ Supplementary Material**

**Supplementary Table 1** Primer sequences of related genes.

| **Gene** | **Forward primer (5' to 3')** | **Reverse primer (5' to 3')** |
| --- | --- | --- |
| H- AGER | GCTCAAAACATCACAGCCCG | ACCTTCCAAGCTTCTGTCCG |
| H-NRP1 | GGTGGGATTGCTGTGGATGA | CAGGTCTGCTGGTTTTGCAC |
| H-β-actin | GGAAATCGTGCGTGACATTAAG | AGCTCGTAGCTCTTCTCCA |

**Supplementary Figure 1** Expression of 5 key genes in different data.

**Figure S2 NRP1 silencing induces oxidative stress and mitochondrial dysfunction in NSCLC cells.** (A) DCFH-DA staining to detect ROS levels. Magnification: 200×. (B) ELISA analysis of MDA levels. (C) JC-1 staining to detect changes in mitochondrial membrane potential, red (aggregates) and green (monomers). Data are presented as mean ± SD. *p<0.05, **p<0.01, ***p<0.001.

**Figure S3 Effects of NRP1 silencing on proliferation, apoptosis, and ROS production in 16HBE cells.** (A) EdU staining analysis of cell proliferation in 16HBE cells following transfection with siRNA-NC or siRNA-NRP1. Magnification: 200×. (B) Flow cytometric analysis of apoptosis in 16HBE cells after NRP1 knockdown. (C) DCFH-DA staining showing intracellular ROS levels in 16HBE cells following NRP1 silencing. Magnification: 200×. Data are presented as mean ± SD from three independent experiments.
